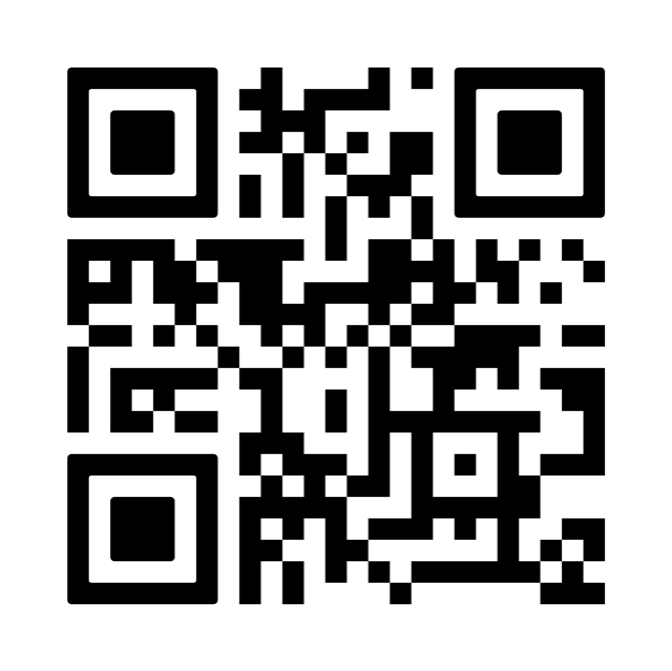# The first robotic-assisted hysterectomy below the bikini line with the Dexter robotic system™

**DOI:** 10.52054/FVVO.16.1.010

**Published:** 2024-03-28

**Authors:** I Alkatout, T Becker, P Nuhn, J Pochhammer, G Peters, K.M. Donald, L Mettler, J Ackermann

**Affiliations:** Department of Gynecology and Obstetrics, Kiel School of Gynaecological Surgery, Kurt-Semm-Center of Minimally Invasive and Robotic Assisted Surgery, University Hospitals Schleswig-Holstein, Campus Kiel, Kiel, Germany; Department of Visceral Surgery, Kurt-Semm-Center of Minimally Invasive and Robotic Assisted Surgery, University Hospitals Schleswig-Holstein, Campus Kiel, Kiel, Germany; Department of Urology, Kurt-Semm-Center of Minimally Invasive and Robotic Assisted Surgery, University Hospitals Schleswig-Holstein, Campus Kiel, Kiel, Germany

**Keywords:** Dexter robotic system, robotic-assisted surgery, hysterectomy

## Abstract

**Background:**

Robotic-assisted hysterectomy (RAH) is a widely accepted minimally invasive approach for uterus removal. However, as RAH is typically performed in the umbilical region, it usually results in scars in cosmetically suboptimal locations. This is the first case of RAH with cervicosacropexy performed below the bikini line, using the new Dexter robotic system™.

**Objectives:**

The aim of this article is to show the surgical steps of the first RAH with cervicosacropexy performed below the bikini line with the new Dexter robotic system™ (Distalmotion), and furthermore assess the feasibility of this approach using this robotic platform.

**Materials and Methods:**

A 43-year-old woman with uterine adenomyosis and recurrent uterine prolapse underwent a robotic-assisted subtotal hysterectomy with cervicosacropexy, performed below the bikini line, using the Dexter robotic system™, at the Clinic of Gynecology and Obstetrics at Universitätsklinikum Schleswig- Holstein (UKHS) in Kiel, Germany.

**Main outcome measures:**

Perioperative data, surgical approach specifics, objective, and subjective outcomes of this new approach.

**Results:**

The procedure was performed without intra-operative complications; estimated blood loss was 10 ml. Operative time was 150 minutes, console time 120 minutes, total docking time 6 minutes. Dexter performed as expected; no device-related issues or robotic arm collisions occurred. The patient did not require pain medication and was released on the second postoperative day.

**Conclusion:**

RAH performed below the bikini line using the Dexter robotic system™ is a feasible, safe, and adequate procedure. These initial results should be confirmed and further extensively refurbished with larger patient cohorts, and functional and psychological outcomes need further investigation.

## Learning objective

The Dexter robotic system™ (Distalmotion, Epalinges, Switzerland) is the new alternative to conventional robotic systems. Thanks to its on-demand concept and the ability to keep a laparoscopic trocar setup, it allows for a new surgical approach for robotic-assisted hysterectomy by operating below the bikini line, with the goal of improving patient cosmetic satisfaction. This video shows the surgical steps of the first robotic-assisted subtotal hysterectomy with cervicosacropexy using the Dexter robotic system™.

## Introduction

Hysterectomy approaches have evolved with technological advancements, from laparotomy to minimally invasive techniques like laparoscopic (LH) and robotic-assisted hysterectomy (RAH) (Alkatout et al., 2016). Since its introduction, several benefits of RAH have been well studied. Compared to LH, RAH offers surgeons robotic precision, high-quality 3D visualisation, surgeon console ergonomics, and tremor-free handling of articulating instruments with fully wristed dexterity (Blavier et al., 2007; Wu et al., 2008).

The Dexter robotic system™ is an open, modular robotic platform introducing the on-demand approach and facilitating efficient switches between conventional and robotic-assisted laparoscopy when needed or desired while keeping a laparoscopic trocar setup. Dexter consists of an open surgeon console, which can remain sterile, two patient carts with instrument arms, and one endoscope arm controlled from the surgeon console, accommodating any 3D-endoscopic system available on-site. Following its clinical approval in 2022, Dexter has been used in urology, general surgery, and gynaecology (Alkatout et al., 2023; Böhlen and Gerber, 2023; Hahnloser et al., 2023; Thillou et al., 2023).

Patient cosmetic satisfaction is becoming increasingly important in gynaecological procedures, especially for young women (Corrado et al., 2018; Elessawy et al., 2020; Goebel and Goldberg, 2014; Mueller et al., 2016). RAH is typically performed in the umbilical region to avoid technical difficulties and collisions of several robotic arms (Gitas et al., 2022), resulting in 8-12 mm scars in cosmetically suboptimal locations. In LH, 5-8 mm instrument trocars are placed in the suprapubic region (Alkatout et al., 2015), allowing for less noticeable scars.

Here, we describe the first subtotal hysterectomy and cervicosacropexy performed below the bikini line using the Dexter system.

## Patients and methods

A 43-year-old woman suffering from dysmenorrhea and hypermenorrhoea due to uterine adenomyosis as well as uterine descent was reviewed in our Clinical Department of Gynecology and Obstetrics at Universitätsklinikum Schleswig-Holstein (UKHS) in Kiel, Germany. She presented with a recurrent genital prolapse in the level I according to DeLancey (1992) after vaginal sacrospinous hysteropexy. The patient weighed 58 kg, had a height of 168 cm, and a body mass index (BMI) of 20.5 kg/m2. She complained of vaginal discomfort, residual urine formation with recurrent urinary tract infections, and constipation.

Comprehensive surgical counseling on the proposed and alternative surgical options was provided to the patient, who selected robotic-assisted laparoscopic subtotal hysterectomy (adenomyosis) with cervicosacropexy. The patient provided written consent to the first robotic-assisted hysterectomy adjoined by cervicosacropexy below the bikini line, i.e. inferior to the line connecting the anterior superior iliac spines (ASIS). Written consent for the subsequent analysis of surgical data was also obtained.

## Results

**Figure 1:**
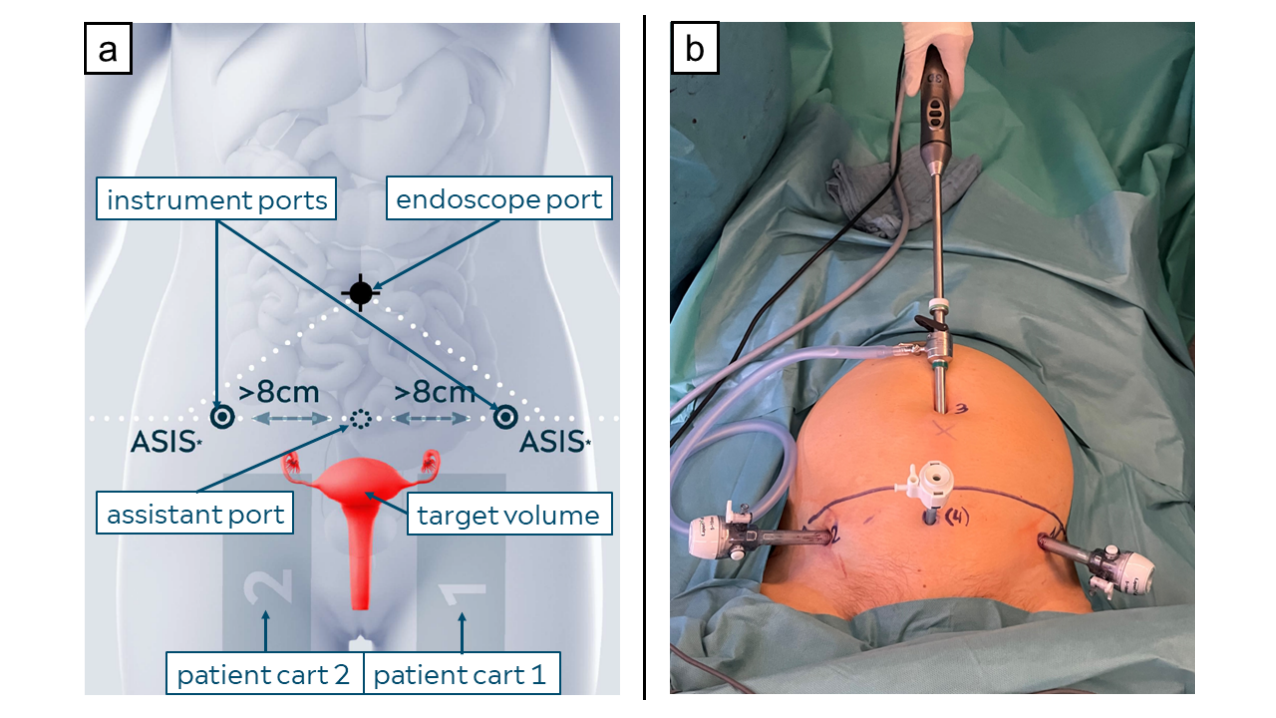
(a) The procedural setup for the hysterectomy below the bikini line; (b) the trocar placement: instrument ports marked with 1 and 2, optical port marked with 3, and assistant port marked with 4.

**Figure 2:**
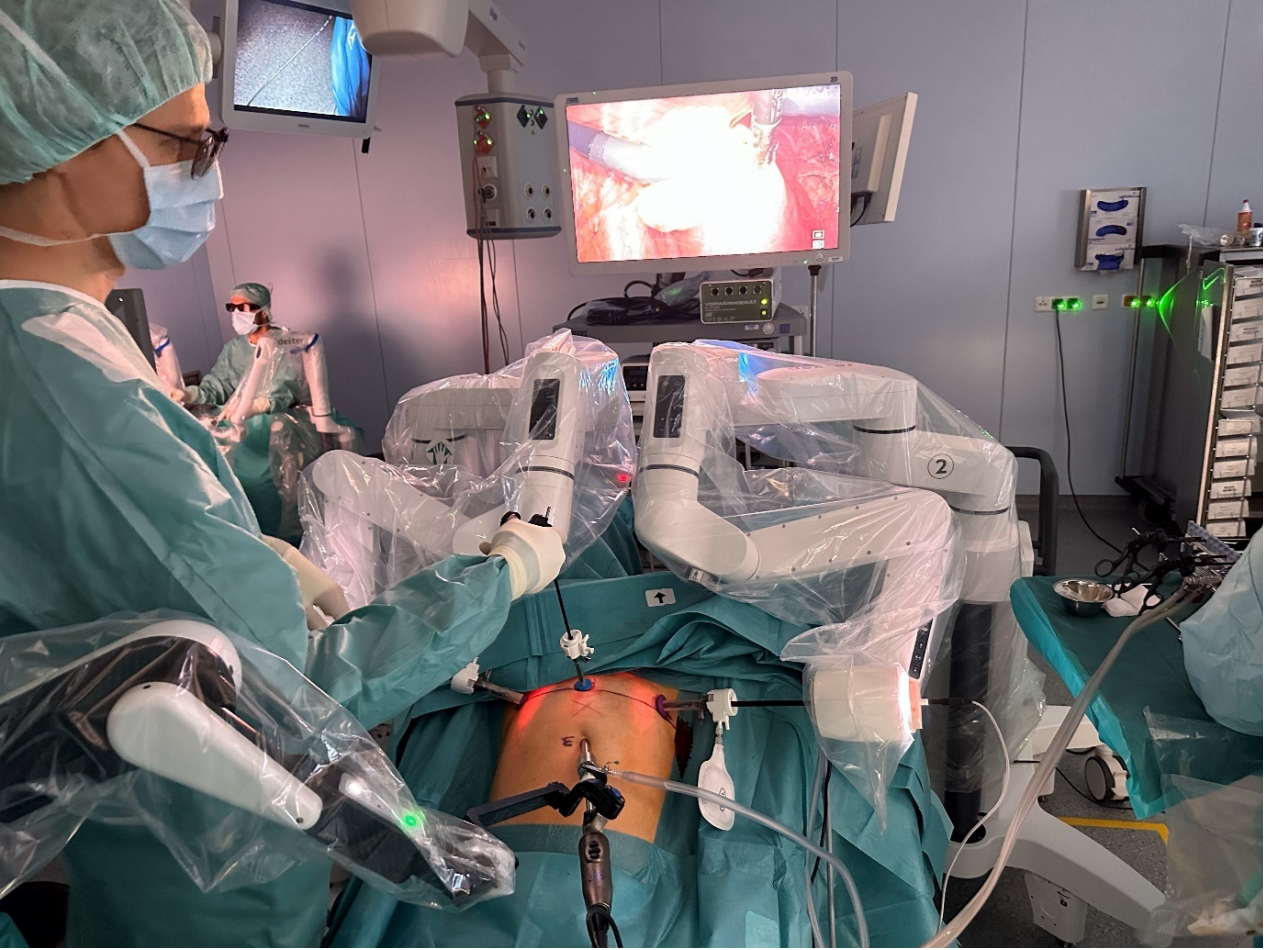
Dexter robotic system during the surgery. From the sterile surgeon console (background upper left quadrant of the photo), the surgeon is controlling the two robotic arms. The assistant surgeon (foreground left) has a sufficient working area between the robot arms.

Prior to the procedure, Dexter was draped with sterile drapes. A 10-mm optical trocar was placed in the umbilicus, pneumoperitoneum was created, and a 10-mm 3D-endoscope with 30° angulation (Karl Storz Endoscopy) was inserted. Two 10-mm translucent trocars were inserted 2 cm medial to each ASIS, 1.5 cm below the ASIS line; a 5-mm assistant-trocar was positioned medially 1.5 cm below the ASIS line (Figure 1). The patient bed was set in a 30° Trendelenburg position. The two patient carts were positioned on each side of the patient bed at the popliteal level, with the instrument arms reaching to the inguinal level (Figure 2). The instrument arms were positioned with a 35° forward angulation and docked to the instrument trocars. The endoscope arm was positioned at the cephalic level and docked to the optical trocar. This configuration of the robotic arms provides access for the surgical team to the patient throughout the entire procedure on both sides of the patient bed. The docking took 6 minutes. The surgical steps are detailed in the video. Robotic instruments included a bipolar Maryland grasper, bipolar Johann grasper, monopolar scissors, and a needle holder. Laparoscopic instruments included a retractor (Alkatout, 2018) (Karl Storz Endoscopy), grasping forceps, a reusable trocar tunneller for introducing the mesh strip (SERAPRO RTD-Ney, Serag Wiessner), and a morcellator (Bowa Medical). The total operative and console times were 150 and 120 minutes, respectively. Dexter performed as expected, without device-related issues or arm collisions. No intraoperative complications were recorded. The estimated blood loss was 10ml. The patient was discharged after two days and did not require pain medication. The postoperative examination demonstrated a physiological elevation of the cervix at -7 cm according to the Pelvic Organ Prolapse Quantification system (POP-Q) with no residual urine.

## Discussion

This is the first RAH below the bikini line, performed using Dexter. The trocar placement utilised in this procedure, with the instrument- and assistant trocars positioned below the bikini line, was previously only feasible using the laparoscopic approach. This set-up is minimising the visible scars. Our experience with the Dexter robotic system demonstrates a highly promising technology, emerging as a distinctive tool for approaches such as RAH under the bikini line.

As demonstrated in Figure 2 and the video, the two Dexter patient carts approach the target volume from behind and extend the robotic arms to reach the target volume from the angles required for the given trocar placement. During the robotic part of the procedure, owing to its unique design, the junior assistant manipulating the uterus can easily access the patient between the patient carts from the caudal side, while from the cranial side, the space left between the robotic arms allows unobstructed patient access to the assistant surgeon, providing them with a clear and spacious working area to operate through the assistant ports without any robotic arm collision. This Dexter feature ultimately enabled efficient and comfortable operating also with the trocar placement below the ASIS line. Consequently, no re-docking of the robotic arms was necessary, and no collision between the robotic arms and the surgical staff occurred during the procedure.

In our case, the laparoscopic assistance was performed by an experienced assistant surgeon in the finalisation of their training. Owing to the quick switch to laparoscopy mode with a single touch of a button, the assistant surgeon performed a seamless transition for the morcellation step, where they purposefully switched to laparoscopy. Thus, a change between the console and the patient bed was not necessary for the surgeon. Nevertheless, thanks to the sterile surgeon console, the surgeon could swiftly stand up and join the assistant in the working area at the patient’s side, to support and coach them in the morcellation process. This is a clear advantage of Dexter compared to other robotic systems. On one hand, the switch between robotic surgery and conventional laparoscopy is relatively easy, and on the other, it is performed quickly due to the surgeon remaining sterile.

Considering that the patient presented with two conditions in need of surgical treatment, symptomatic uterine adenomyosis and uterine descent, the combination of laparoscopy and robotic surgery is ideal. In contrast to urology and visceral surgery, the surgeons in gynaecology are not necessarily dependent on a robot-assisted system. However, due to the seven degrees of freedom of the instruments, robotic assistance enables easier dissection and endoscopic suturing, especially in difficult-to-access surgical areas. Therefore, this possibility of switching between conventional laparoscopy and robotic surgery seems excellent for complex cases like in urogynaecology.

From a cosmetic aspect, the described Dexter hysterectomy setup allowed for the placement of both instrument ports and the assistant port below the ASIS line, thus leaving the patient with a more favourable scar location compared to the scars typically associated with RAH using other robotic platforms (Alkatout et al., 2015; Borse et al., 2022; Gueli Alletti et al., 2022; Monterossi et al., 2022; Puntambekar et al., 2021).

## Conclusion

To conclude, RAH performed below the bikini line using the Dexter robotic system is a feasible, safe, and adequate procedure with supporting evidence indicated by total operative time, complication rate, intraoperative blood loss, length of hospital stays, and patient cosmetic and overall satisfaction. The quick switching opportunities between conventional laparoscopy and rigid console surgery is the eagerly awaited bridging option between the two camps. These initial results should be confirmed and further extensively assessed with larger patient cohorts, and the functional and psychological outcomes need further investigation.

## Video scan (read QR)

https://vimeo.com/920372026/1c0f3ea89b?share=copy

**Figure:**